# One Step forward: Benthic Pelagic Coupling and Indicators for Environmental Status

**DOI:** 10.1371/journal.pone.0141071

**Published:** 2015-10-23

**Authors:** Panagiotis D. Dimitriou, Nafsika Papageorgiou, Christos Arvanitidis, Georgia Assimakopoulou, Kalliopi Pagou, Konstantia N. Papadopoulou, Alexandra Pavlidou, Paraskevi Pitta, Sofia Reizopoulou, Nomiki Simboura, Ioannis Karakassis

**Affiliations:** 1 University of Crete, Biology Department, 70013 Heraklion, Crete, Greece; 2 Hellenic Centre for Marine Research, 71013 Heraklion, Crete, Greece; 3 Hellenic Centre for Marine Research, 19013 Anavyssos, Greece; Auckland University of Technology, NEW ZEALAND

## Abstract

A large data set from the Eastern Mediterranean was analyzed to explore the relationship between seawater column variables and benthic community status. Our results showed a strong quantitative link between the seawater column variables (Chlorophyll *a* and Eutrophication Index) and various indicators describing benthic diversity and community composition. The percentage of benthic opportunistic species increased significantly in the stations with high trophic status of the seawater column and so did the strength of the coupling between values of seawater column and benthic indicators. The Eutrophication Index threshold level of 0.85, separating the “Bad and Poor” from “Moderate to High” conditions could serve as an acceptable critical value above which there is a readily observable change in benthic community composition.

## Introduction

Today it is understood that eutrophication is one of the prominent anthropogenic vectors that changes the state of all aquatic ecosystems from the Arctic to the Antarctic [1], with severe ecological and economic consequences. Among a number of definitions for eutrophication (Kitsiou & Karydis [2] reviewed in Karydis [3]), a definition suggested by Ferreira, Andersen (4] and adopted by the European Union proposes that eutrophication is the increased growth/production that results from enhanced nutrient input especially nitrogen and/or phosphorus. Furthermore, the diagnosis must be confirmed by an 'undesirable disturbance' to the balance of organisms and seawater quality that may follow from increased production (increased growth, primary production and biomass of algae). It has been subsequently argued [5–7] that research on marine coastal eutrophication, despite some recent successful examples, is still in its infancy and there is room for a new paradigm [8] based on the interaction of the components of the highly complex coastal marine ecosystem.

In the context of the Water Framework Directive (WFD) and European Marine Strategy Framework Directive (MSFD), a series of indicators focusing on seawater column or benthos have been used to define the Ecological Status of the coastal areas [9]. However, there has been little effort to co-examine the interaction between these two components of the ecosystem, which would be expected to co-vary in a wide range of anthropogenic pressures. Kitsiou & Karydis [2] argued that, although there is a tendency to increase the number of variables used for quantitative assessment of eutrophication, it is doubtful whether they are all necessary in assessment studies. They concluded that the number of variables needed is rather limited: inorganic nitrogen (nitrate, nitrite, and ammonia), inorganic phosphorus (orthophosphates) and production of organic matter (Chlorophyll *a* (Chl-*a*), phytoplankton cell number and macrophyte biomass).

Many indicators have been proposed for the classification of coastal seawater bodies into oligotrophic, mesotrophic and eutrophic, based on their trophic status (reviewed by Karydis [3]). Abiotic indices are usually based on nutrient concentrations; for example, the N/P ratio [10] or nutrient algorithms [11] or specific nutrient variables [12–14]. A eutrophication scale based on Chl-*a* concentration was first proposed by Karydis [15] and was used extensively for the Greek seas. It included four levels of eutrophication: eutrophic, higher mesotrophic, lower mesotrophic, and oligotrophic. This scale was later modified by Simboura et al. [16] to comply with the five levels of ecological status implied by the WFD. The proposed scale based on Chl-*a* concentrations is: <0.1 μg L^-1^ High, 0.1–0.41 μg L^-1^ Good, 0.4–0.61 μg L^-1^ Moderate, 0.6–2.211 μg L^-1^ Poor and >2.211 μg L^-1^ Bad.

Two multimetric indices have been proposed during the past ten years: the Trophic Index (TRIX) [17] and the Eutrophication Index (EI) [18]. The formula of TRIX is a linear combination of the logarithm of four variables: Chl-*a*, dissolved inorganic nitrogen (DIN), total phosphorus (TP) and the absolute percentage of deviation from oxygen saturation, with a five-level scale added by Pettine et al. [19] for Italian seawater bodies. The EI is another multimetric index calculated using nutrient (nitrite, nitrate, ammonia, phosphorus) and Chl-*a* concentrations, and also uses a five-level scale. It was found efficient in discriminating eutrophic levels characterizing oligotrophic, mesotrophic and eutrophic conditions. Ferreira et al. [4] provided an extended review on eutrophication indicators and monitoring capabilities in the context of the MSFD.

On the other hand, benthic macrofauna is an excellent ecosystem component which mirrors the ecological status of the marine environment, and therefore it has become a standard component of marine environmental monitoring [20]. A number of benthic ecological indicators have been proposed in the past 12 years as a means for assessing disturbance of the benthic environment. Among these, AZTI Marine Biotic Index (AMBI) [21], Multivariate-AMBI [22], Benthic Index (BENTIX) [23], Benthic Quality Index (BQI) [24] as modified in Leonardsson et al. [25] and the Shannon Diversity index H’ [26] are the most widely used. More recently, indicators based on higher taxonomic levels such as the Benthic Opportunistic Polychaeta Amphipoda index (BOPA, [27] and the BQI-Family index [28] have been discussed as potential tools for the implementation of the WFD (e.g., [4, 29–41]) in different disturbance/pollution gradients, geographical regions and benthic habitats. Although all the above indices use different methods, they are all based on the well-known paradigm of benthic succession along gradients of organic enrichment [42] and they aim to classify the Ecological Status of a certain marine ecosystem into the five-level scale imposed by the WFD.

There is little doubt that benthic life depends on pelagic processes. However, there have been complications when attempting to develop quantitative descriptions of benthic pelagic coupling [43]. Up to now, various models relating pelagic primary production to oxygen demand in sediments [44–47], organic matter content in sediments [48, 49], or biogeochemical sediment variables [50] have been proposed. However, no quantitative relationship has been established between the variables in the seawater column and the ecological condition of the sediment as described by diversity or ecological quality indices. An exception to this was the CSTT [51] study, which suggested that a concentration of chlorophyll (10 mg m^-3^ during the summer months) in the seawater column could be used as a threshold imposing degradation in Scottish lochs. This value has been cited/reproduced in the literature (see Tett [52]) without either confirmation or falsification from other relevant studies. Similarly, it has been suggested that the hypoxia threshold of dissolved oxygen concentration in the seawater column, assumed to be 2 mg L^-1^ by Diaz [53], may vary considerably among different benthic organisms [44] and therefore it cannot be adequately captured by a single universal threshold.

As stated above, the benthic indicators are widely trusted since they reflect and integrate the environmental pressures in a given site. When the major factor affecting the environmental quality is eutrophication, it would be expected that the geochemical variables in the seawater column are driving the benthic system indicators towards the low quality end values. In this context it would be useful to know what levels of eutrophication (and related variables) result in “unacceptable” conditions regarding the benthos; therefore, it is reasonable to find out whether there is a dose-response curve connecting the seawater column variables or indicators with the condition of the benthic communities. In addition, it would be useful to know if there is a threshold in these seawater column variables beyond which the benthic community becomes invariably degraded.

The aim of the present paper was to analyze a large data set from a generally oligotrophic environment to explore the relationship between seawater column variables and benthic community status. The hypotheses tested here are that (a) changes in seawater column trophic status affect species composition patterns of benthic macrofaunal communities and, therefore, (b) changes in seawater column trophic status affect the values of benthic indicators and the resulting assessment of Ecological Status.

## Methods

### Dataset description

A series of data sets were used, comprising samples collected from stations located in different seas of the Eastern Mediterranean basin as the result of various scientific and monitoring programs. No specific permissions were required for these locations/activities. The field studies did not involve endangered or protected species. Sampling locations, dates and sampling information are provided in S1 Table. The condition on which these data sets could be used for the analysis was that both benthic macrofauna and seawater column had been sampled simultaneously. Seawater column variables had to include measurements of Chl-*a* concentration as well as inorganic nutrients concentration (PO_4_^3–^, NO_3_^–^, NO_2_^–^ and NH_4_^+^) at the bottom layer of seawater. Dissolved oxygen (DO) concentration, sediment total organic carbon (TOC), redox potential (Eh), sea bottom depth (Depth) and the sediment silt and clay content (% Silt & Clay) data, wherever available, were also used. All samples were collected from coastal areas where the main source of the organic matter supplied to the seabed was phytoplankton precipitation. Consequently, samples taken in the vicinity of allochthonous organic matter sources such as aquaculture, ports, sewage or industrial wastes and other forms of disturbance such as dredging or heavy metal toxicity were excluded. The dataset used in the present study includes published data [34, 35, 54–64], as well as unpublished data from the University of Crete and the Hellenic Center of Marine Research (HCMR). Overall, it included 126 sampling stations; 34 in the Ionian Sea, 68 in the Northern Aegean Sea and 24 in the Southern Aegean or Cretan Sea. Data sets for abundances of benthic species were accepted for the data analysis on the condition that three to five replicates were collected from each sampling station.

Seawater samples were collected using Niskin bottles. Chl-*a* measurements were carried out by seawater filtration (GFF 0.7 μm) and fluorometer measurement, DO by Winkler method or calibrated Conductivity–Temperature–Depth instruments, and nutrient measurements were carried out with the appropriate chemical protocols. TOC was determined from triplicate core tubes (4.5 cm internal diameter) by means of a CHN Analyzer following the procedure of Hedges and Stern (1984) and Eh was measured in core samples at 2–4 cm intervals from the seawater–sediment interface by means of an electrode standardized with Zobell’s solution. The % Silt & Clay content was measured by sieving. Detailed information about locations, dates, depths, and sampling gears can be found in S1 Table.

### Diversity and Biotic indices

All taxonomic names included in the accepted data sets were checked for synonyms by means of the TaxonMatch online tool of the World Register of Marine Species portal (http://www.marinespecies.org/aphia.php?p=match). The abundance of benthic species was used to calculate a number of established diversity indices such as total number of species (S), total number of individuals (N), Hurlbert’s ES(10), and Shannon *H*’ (log_2_) using the PRIMER 6.1 software [65]. In addition, a series of biotic indices widely used in the context of the WFD were calculated. In all cases the formulas provided by the authors have been followed, as described in the respective publication: for M-AMBI [22], the calculation program, using default settings and species inventory provided in the authors’ web-site (http://ambi.azti.es) dated 3/2012; for BENTIX [23], the excel add-in provided at http://www.hcmr.gr/gr/listview3.php?id=1195; in the cases of BQI [24] as modified in Leonardsson et al. [25] and BQI-Family [28], the Biological Indices Calculation Tool (BICT) online calculation tool hosted at Lifewatch Greece website (http://www.lifewatchgreece.eu) was used with the species or families tolerance values (ES50_0.05_) list provided by [28] at (http://www.sciencedirect.com/science/article/pii/S1470160X12000544#MMCvFirst). Eutrophic Index (EI) [18] was also calculated using the instructions provided by the authors. The EI classifies the seawater body in five Ecological Status levels: (a) less than 0.04, (b) 0.04–0.38, (c) 0.38–0.85, (d) 0.85–1.51, and (e) greater than 1.51, corresponding to the five Ecological Status levels used in the WTD: High, Good, Moderate, Poor, Bad.

All the variables used are presented in Table 1 and the distribution of sampling stations among Ecological Status categories in EI, Chl-*a*, BQI-Family and BENTIX are shown in Table 2.

**Table 1:** List of variables used in the present study. Chl-*a*, chlorophyll *a*; EI, Eutrophic Index; DO, dissolved oxygen; TOC, total organic carbon; Eh, redox potential; S, number of species; ES(10), expected number of species for 10 individuals; BQI, Benthic Quality Index; BQI-Family, Benthic Quality Index–Family; *H’*, Shannon Diversity Index; BENTIX, Benthic Index; M-AMBI, Multivariate AZTI Marine Biotic Index.

Abiotic variables		Biotic variables	
Seawater Column	Sediment	Diversity	Biotic indices
Chl-*a*	TOC	S	BQI
PO_4_^3–^	Eh	ES(10)	BQI-Family
NO_3_^–^	% Silt & Clay		*H’*
NO_2_^–^	Depth		BENTIX
NH_4_^+^			M-AMBI
EI			
DO			

**Table 2:** Number of sampling stations in each Ecological Status category according to four seawater column and benthic indices. Chl-*a*, chlorophyll *a*; EI, Eutrophic Index; BQI-Family, Benthic Quality Index—Family; BENTIX, Benthic Index.

	EI	Chl-*a*	BQI-Family	BENTIX
Bad	15	20	15	0
Poor	17	27	17	18
Moderate	40	26	19	28
Good	54	41	76	55
High	0	12	0	25

Borja et al. [33] suggested that there is a need to set correct reference conditions common for all the indicators used in the WFD context for the sound assessment of Ecological Status. Out of all indices used in the present study, the BQI-Family and BENTIX have been calibrated using stations and reference conditions from the Eastern Mediterranean. The BQI-Family had, in general, better correlations to environmental variables than BENTIX. Consequently, in analysis requiring Ecological Status classification, stations were labeled according to the Ecological Status of the BQI-Family index. Benthic species were categorized according to their ES50_0.05_ values using the species tolerance list provided by Dimitriou et al. [28] into three categories: opportunistic (ES50_0.05_ 1–10), intermediate (ES50_0.05_ 10–20), and sensitive (ES50_0.05_ 20–30).

### Statistical Analyses

For all the analyses, species abundance data were square root transformed. Analysis of similarity (ANOSIM) was performed to detect differences in the benthic community structure between the different Ecological Status categories of the seawater column, as indicated by EI or Chl-*a* scale. When necessary, Analysis of Variance (ANOVA) or Spearman Correlation was performed using the SPSS 21 program. Furthermore, two ordination techniques were performed: (i) Nonmetric Multidimensional scaling (MDS) with the Bray-Curtis similarity index, using Primer 6.1, to analyze variations in community composition in relation to the trophic status of the seawater column and with the Ecological Status, as calculated by the application of the BQI-Family index; and (ii) Canonical Correspondence Analysis (CCA), using CANOCO 4.5 for Windows [66] to assess the effect of environmental variables on benthic community structure. The CCA method was selected based on the length of the gradient calculated by detrended correspondence analysis (DCA)[67]. Since the first DCA axis had a gradient length equal to 6.387 standard-deviation units, the use of a unimodal ordination technique was justified. Prior to the CCA analysis, each environmental variable was tested using the variance inflation factor (VIF) to identify useless constraints. A VIF value over 10 indicates that the variable is highly correlated with the other variables and therefore needs to be excluded from the analysis. In a CCA plot, the arrows for environmental variables point in the general direction of maximum environmental change across the diagram, and their lengths are approximately proportional to the rate of change in that direction[67]. The projection of a variable on the environmental vector is an approximation of the “optima” regarding that particular environmental variable [67]. The relationship between two variables can be examined based on the angle between their two arrows; an angle smaller than 90° indicates a positive relation between the two variables; the smaller the angle, the closer the positive relation of the two variables. An angle between 90° and 180° suggests a negative correlation. Finally, there is no relation between two variables when their angle is 90° [67].

To test the effect of seawater column eutrophication on the relative abundance of macrobenthic opportunistic species (defined as those having ES50_0.05_ < 10), the percentage of benthic opportunistic species in each station was calculated. Stations, were subsequently grouped according to the Ecological Status of the seawater column as indicated by the Chl-*a* scale or the EI index, and the average percentage of benthic opportunistic species for each Ecological Status level was plotted.

To further explore the relationship between benthic and seawater column indices we divided the data set into “acceptable” (“High” and “Good”) and “unacceptable” (“Bad” to “Moderate”) in terms of benthic indicators. For each of these two groups we carried out a Spearman correlation between values of the seawater column (EI or Chl-*a*) and benthic (BENTIX and BQI-Family) indices.

## Results

**Fig 1:**
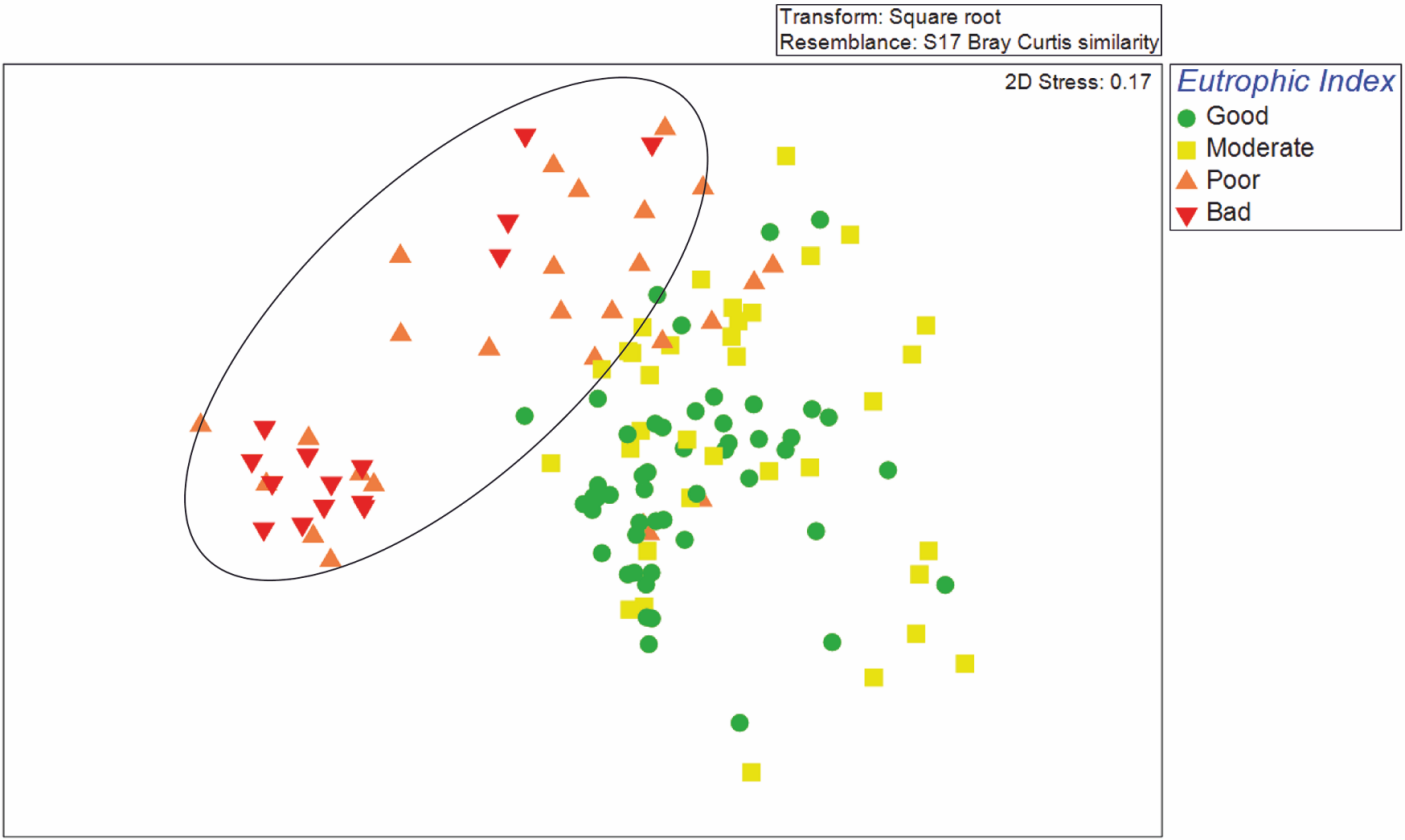
Non-metric multi dimensional scaling (nMDS) plot of macrobenthic data but with stations labeled after the Ecological Status defined by means of the Eutrophic Index in the overlaying water column.

The ANOSIM test showed statistically significant differences in the benthic macrofaunal community composition between all possible pairs of Eutrophic Index–Ecological Status and in most cases of Chl-*a*–Ecological Status of the seawater column in the respective sampling stations (Table 3). The MDS plot (Fig 1) was based on macrobenthic species-abundance data, but the stations were classified into groups according to the Ecological Status of the seawater column, as indicated by the EI index. Sampling stations with “Poor” or “Bad” Ecological Status under the EI are grouped together in the upper left corner of the MDS plot, whereas those of “Moderate” or “Good” Ecological Status are scattered in the rest of the plot.

**Table 3:** Results of ANOSIM test of the benthic community data. Stations were grouped after the status of the seawater column based on the Eutrophic Index and Chl-*a*.

Eutrophic Index				Chl-*a*				
	Bad	Poor	Moderate		Bad	Poor	Moderate	Good
**Poor**	*	-		**Poor**	**	-		
**Moderate**	**	**	-	**Moderate**	**	ns	-	
**Good**	**	**	**	**Good**	**	**	ns	-
				**High**	**	ns	**	ns

**: p < 0.01,

*: p < 0.05,

ns = non significant.

**Fig 2:**
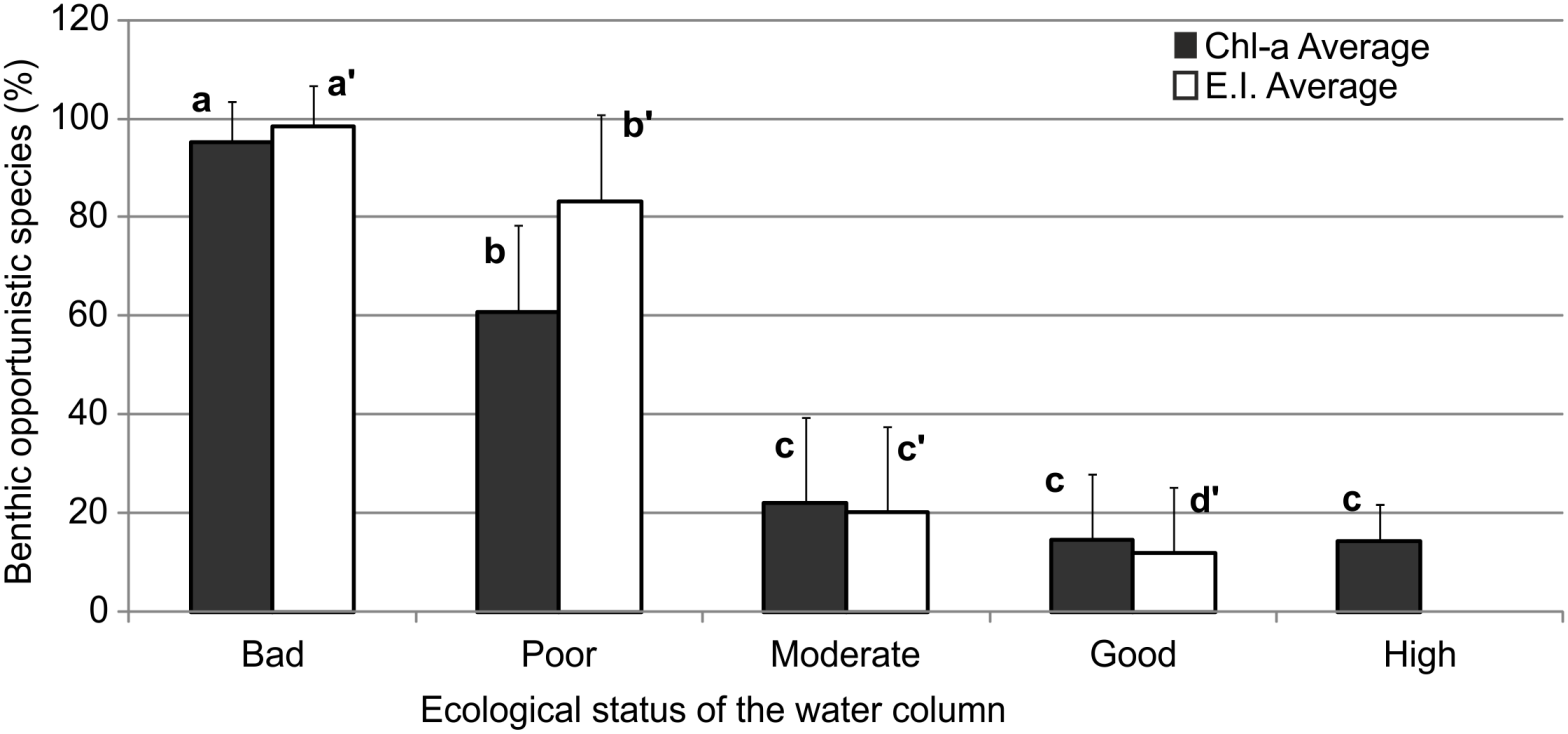
Average percentage (± SD) of benthic opportunistic species (ES50_0.05_ < 10) in each Ecological Status of the water column as indicated by the Chl-*a* scale or the Eutrophic Index. Statistically significant differences between groups (p < 0.05) in the post hoc Tukey tests for each ANOVA test are indicated by differences within the following groups of letters: Chl-*a* (a–c) and EI (a’–d’).

The average percentage of benthic opportunistic species for every EI or Chl-*a* group is presented in (Fig 2). The percentage significantly differed (ANOVA p < 0.05) among stations with “Bad”, “Poor” and “Moderate” Ecological Status for both Chl-*a* and EI whereas in the case of EI there was also a significant difference between “Moderate” and “Good” Ecological Status. At the threshold value separating the seawater column “Moderate” from “Poor” Ecological Status, a shift in the percentage of benthic opportunistic species occurs, with values ranging between 21 and 61% for the Chl-*a* scale and between 20 and 83% for EI, reaching almost 100% at the “Bad” Ecological Status.

The effect of all variables taken into account in the CCA was statistically significant (p < 0.002); however, NO_2_^–^ had a VIF > 10 and therefore was excluded from the analysis; this was also the case for Eh, which had many missing values. The results of the CCA (Table 4) showed that Axis 1 accounts for 0.857 variance and Axis 2 for 0.51. The correlations between the species abundance values and those of the environmental variables were high (0.968 and 0.944 for the two axes). Furthermore, the first two axes explained 26.2% of the total species variance and 63.8% of the species environmental variance, which is a sufficiently high percentage for a data set of this size [67]. Finally, the results of the Monte-Carlo permutation test (using the default 499 permutations) showed that the analysis was statistically significant.

**Table 4:** Canonical Correspondence Analysis results.

**Axes**	**1**	**2**	**3**	**4**	**Total inertia**
Eigenvalues	0.857	0.51	0.388	0.272	8.459
Species-environment correlations	0.968	0.944	0.933	0.91	
Cumulative % of explained variance					
of species data	10.1	26.2	30.7	34	
of species-environment relation	33.7	63.8	79.1	89.8	
Sum of all eigenvalues	8.459				
Sum of all canonical eigenvalues	2.541				
**Monte-Carlo permutation test**		**Eigenvalue**		**F-ratio**	**P-value**
Test of significance of first canonical axis		0.857		4.285	0.002
Test of significance of all canonical axes		2.541		2.33	0.002

**Fig 3:**
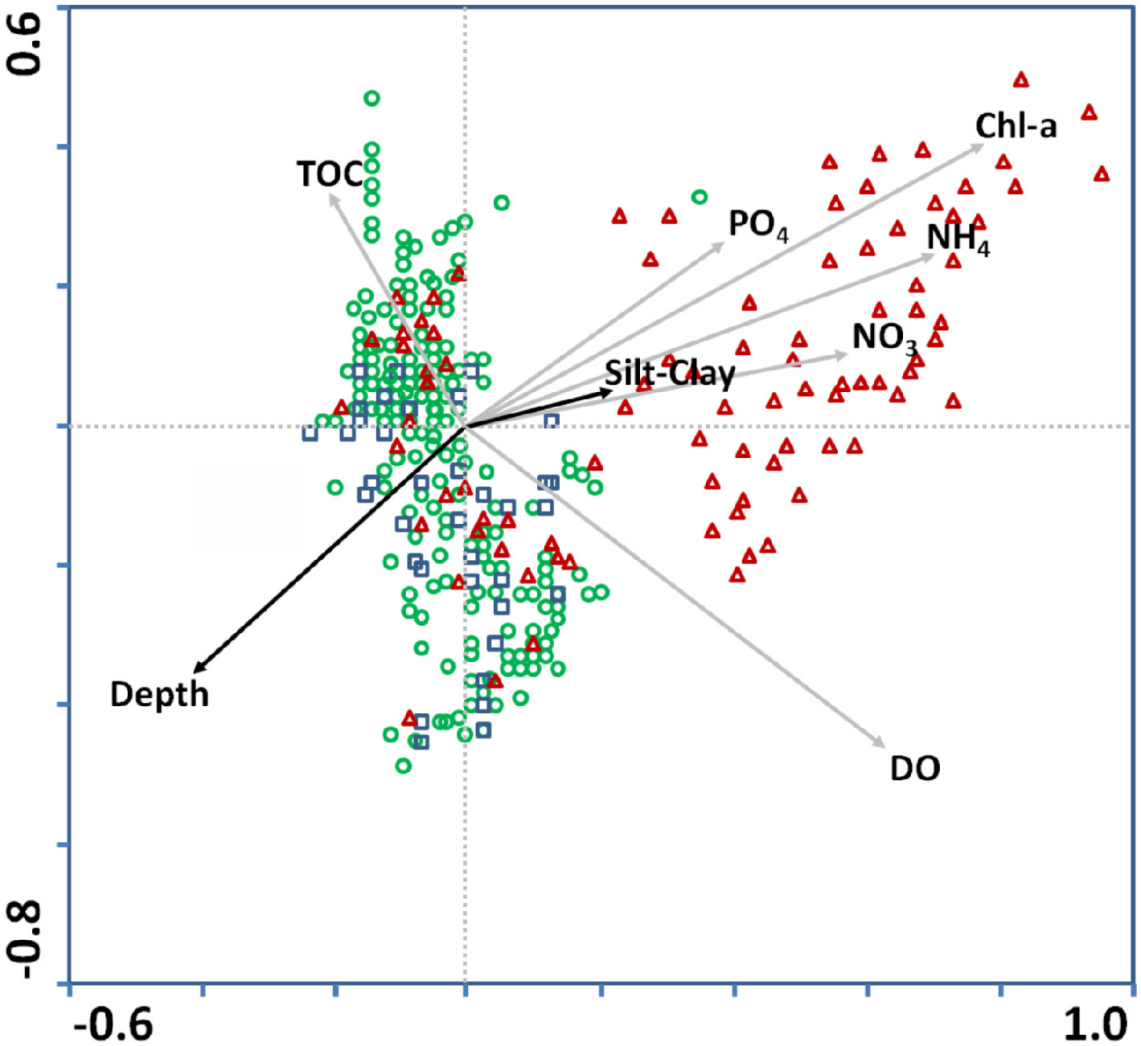
Canonical Correspondence Analysis (CCA) biplot with species and environmental values. Triangle represents species with ES50_0.05_ of 1–10, circle 10–20, square 20–30. Dark and gray arrows represent sediment and water column variables.

In the CCA biplot (Fig 3) depicting the benthic species and environmental variables, species were separated into three categories; i.e. opportunistic, intermediate and sensitive. The position of Chl-*a*, PO_4_^3–^, NO_3_^–^ and NH_4_^+^ arrows indicate a strong positive correlation between those variables. The higher the values, the more the opportunistic species occur. The DO concentration also plays a major role, as indicated by the length of its arrow, but it is not closely associated with a group of species. For the sediment variables, TOC is associated with species of intermediate ES50_0.05_ values, Depth is not associated with any of the species group and % Silt & Clay plays a less important role compared to the other variables in the present data set.

The results of Spearman's correlation coefficient calculated between the geochemical and biological variables and indices (Table 5) showed highly significant correlation between Chl-*a* and almost all benthic variables (p < 0.01), positive in the case of silt content of the sediment and negative with all the indices related to diversity or ecological quality. The EI (highly correlated with Chl-*a*) showed the same correlation pattern with Chl-*a* and stronger correlations with most benthic indices. The DO in the seawater above the seabed was positively correlated with Chl-*a* and EI. On the other hand, the Eh was negatively correlated to the Chl-*a* and EI and also with the biotic indices.

**Table 5:** Spearman rank correlation between biological and geochemical variables and/or indices. Chl-*a*, chlorophyll *a*; EI, Eutrophic Index; DO, dissolved oxygen; TOC, total organic carbon; Eh, redox potential; S, number of species; ES(10), expected number of species for 10 individuals; BQI, Benthic Quality Index; BQI-Family, Benthic Quality Index–Family; *H’*, Shannon Diversity Index; BENTIX, Benthic Index; M-AMBI, Multivariate AZTI Marine Biotic Index.

	Chl-*a*	NH_4_^+^	PO_4_^3–^	NO_3_^–^	NO_2_^–^	EI	DO	TOC	Eh	% Silt & Clay
**PO**_**4**_^**3–**^	ns	ns								
**NO**_**3**_^**–**^	ns	0.33**	0.24**							
**NO**_**2**_^**–**^	ns	0.24**	0.33**	0.61**						
**EI**	0.73**	0.54**	0.29**	0.50**	0.55**					
**DO**	0.67**	0.35**	-0.52**	0.29*	-0.19	0.36**				
**TOC**	ns	ns	ns	ns	ns	ns	ns			
**Eh**	-0.68**	-0.58**	ns	ns	ns	-0.61**	ns	-0.66**		
**% Silt & Clay**	0.39**	0.31**	0.41**	ns	0.35**	0.37**	ns	0.55**	-0.66**	
**BQI**	-0.49**	-0.40**	-0.18*	-0.18*	-0.29*	-0.63**	-0.46**	-0.24*	0.70**	-0,27*
**BQI-Family**	-0.60**	-0.40**	ns	-0.14*	-0.24*	-0.65**	-0.46**	-0.25*	0.67**	-0,28*
***H’***	-0.57**	-0.40**	ns	ns	ns	-0.54**	-0.40**	-0.12*	0.73**	ns
**BENTIX**	-0.34**	ns	ns	ns	ns	-0.33**	ns	-0.20*	0.38**	ns
**M-AMBI**	-0.58**	-0.38**	-0.26**	-0.20**	-0.33**	-0.67**	-0.48**	-0.15*	0.69**	-0,31**
**S**	-0.34**	-0.45**	-0.35**	-0.23**	-0.37**	-0.61**	-0.28*	ns	0.66**	-0,41**
**ES(10)**	-0.54**	-0.42**	ns	-0.12*	-0.30**	-0.63**	-0.44**	-0.36**	0.75**	-0,36**
**Depth**	-0.60**	-0.38**	0.29**	ns	ns	-0.37**	-0.74**	ns	0.64**	ns

**Fig 4:**
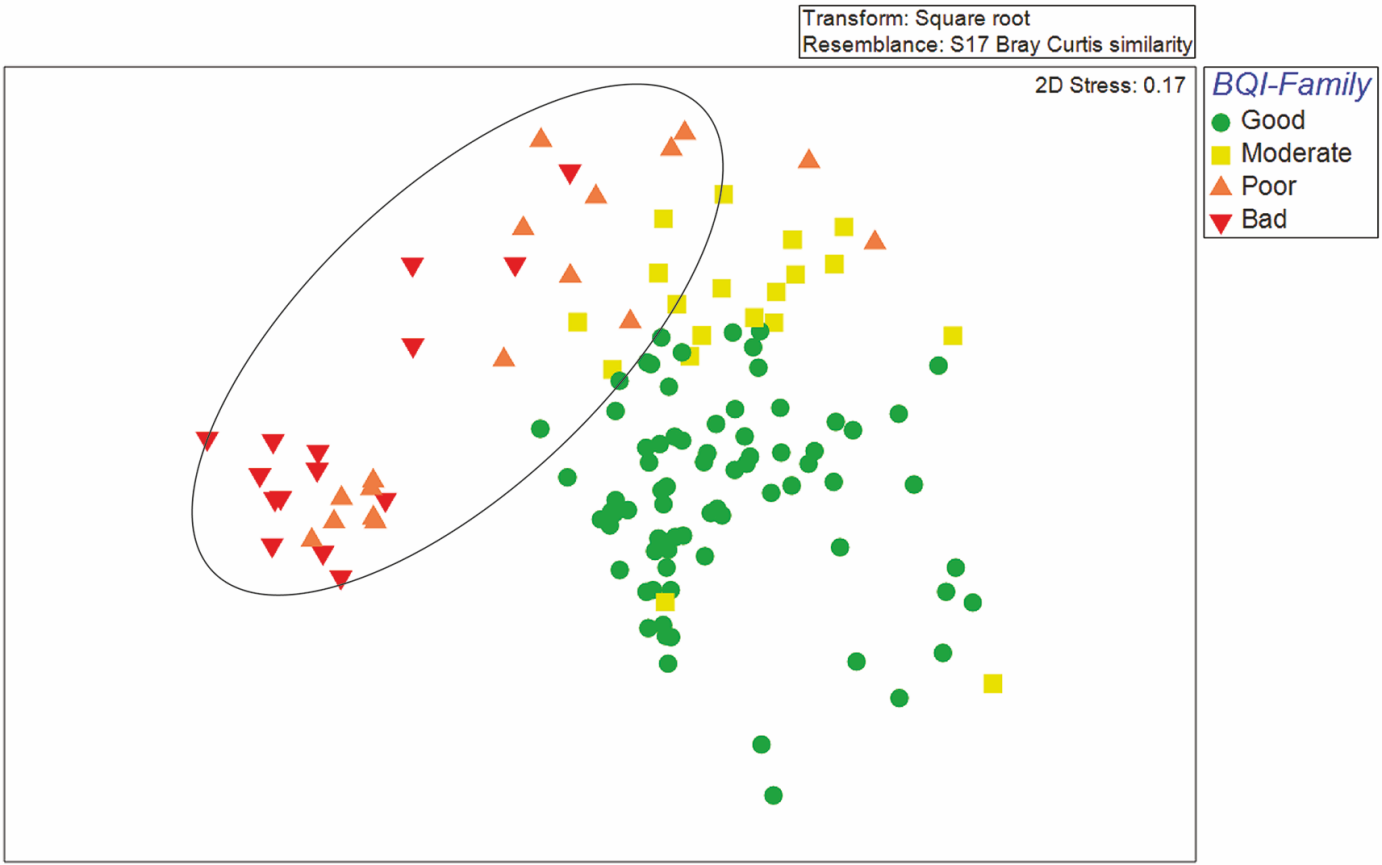
Non-metric multi dimensional scaling (nMDS) plot of macrobenthic data with stations labeled after the Ecological Status defined by means of the Benthic Quality Index—Family index. The ellipse surrounds the stations with “Bad” or “Poor” Ecological Status in the water column as indicated in Fig 1.

* p < 0.05,

** p < 0.001,

ns = non significant,

The MDS plot in Fig 4 was based on macrobenthic species-abundance data (as in Fig 1), but the stations here were labeled after the Ecological Status of the BQI-Family index. Sampling stations with “Poor” or “Bad” Ecological Status under the BQI-Family are grouped together in the upper left corner of the MDS plot, whereas those of “Moderate” or “Good” Ecological Status are scattered in the remaining plot space. There is a remarkable similarity between the two figures (Figs 1 and 4) regarding the distribution of the ecological quality status.

When separating the samples into “acceptable” and “unacceptable” in terms of the benthic indices (Table 6) there is a strong correlation in the “unacceptable” stations (p < 0.001) between all four combinations of Chl-*a*, EI and BENTIX, BQI-Family, whereas in the “acceptable” stations the correlations were weaker (p < 0.05) and were significant only between Chl-*a*–BENTIX and EI–BQI-Family.

**Table 6:** Results of Spearman correlation analysis between water column and benthic indices for two groups of stations based on the Ecological Status of the benthic indicators, i.e. “acceptable” (“High” or “Good”) and “unacceptable” (“Bad”, “Poor” or “Moderate”). Chl-*a*, chlorophyll *a*; EI, Eutrophic Index; BQI-Family, Benthic Quality Index–Family; BENTIX, Benthic Index.

	Chl-*a*		EI	
	BENTIX	BQI-Family	BENTIX	BQI-Family
**Acceptable**	-0.26*	ns	ns	-0.31*
**Unacceptable**	-0.55**	-0.68**	-0.50**	-0.84**

**Fig 5:**
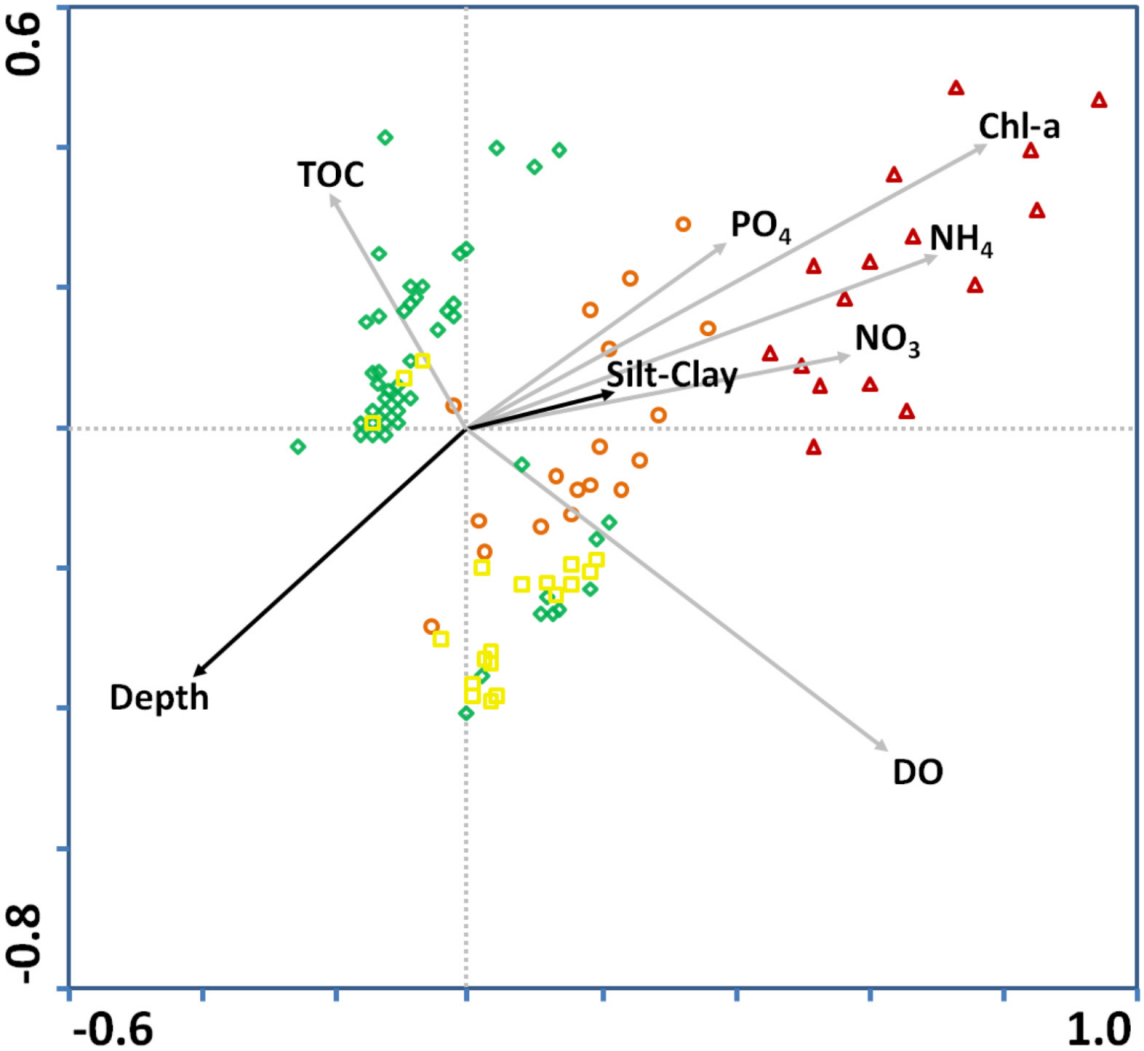
Canonical Correspondence Analysis biplot with stations and environmental variables. Stations are labeled after the Ecological Status defined by means of the Benthic Quality Index—Family index: Symbols indicate Ecological Status: triangle, Bad; circle, Poor; square, Moderate; diamond, Good.

* p < 0.05,

** p < 0.001,

ns = non significant,

From the second CCA biplot with stations and environmental variables, it can be concluded that stations classified as “Bad” Ecological Status are closely related to Chl-*a* and nutrients, while those with “Poor” and “Moderate” Ecological Status are more related to DO and Depth. Stations with “Good” Ecological Status are closely related to TOC content (Fig 5).

## Discussion

The results of the present study demonstrate that there is a remarkably tight link between the trophic status of the seawater column and the benthic macrofaunal community composition. Although the coastal environment is considered to be relatively unstable in terms of the seawater column variables, particularly in exposed marine sites [68], the analysis showed that changes in the seawater column environmental variables are readily (and quantitatively) reflected by the underlying benthic communities. The categorization of the benthic samples according to the EI seemed more efficient than that based on the Chl-*a* concentration. However, it should be noted that there is not always consensus among experts regarding the classification of samples between consecutive ecological quality categories [69] as no single class of indicators consistently outperforms all other classes of indicators [70]. The change in community structure induced by changes in the seawater column trophic status is related to the increase in the proportion of opportunistic benthic species. Such increase was found when crossing the threshold between “Moderate” and “Poor” Ecological Status in the seawater column but less conspicuously (although significant in the case of EI) when crossing the threshold between “Moderate” and “Good”. Here, the opportunistic species are defined as those with ES50_0.05_ < 10 (index introduced by Rosenberg et al. [24]), i.e. those species which are able to tolerate adverse environmental conditions resulting in low benthic diversity according to the benthic succession paradigm [42].

The CCA results suggest that a set of environmental gradients affects the distribution of species with different strategies. The most significant variables are Chl-*a* and nutrients, DO, Depth and TOC. The abundance of opportunistic species is closely related to the Chl-*a* and nutrients whereas the intermediate and sensitive ones are associated with a combination of DO, Depth and TOC in sediments.

Both Chl-*a* and EI were highly correlated with benthic indices and geochemical variables. The Spearman correlation coefficients of Chl-*a* and EI were similar in the case of Eh, BENTIX, Shannon (although it has been argued [71] that the Shannon index sometimes is unable to explain a significant amount of variability) and % Silt & Clay but EI showed much stronger correlations in the case of BQI, M-AMBI, BQI-Family, S and ES(10). On the other hand, Chl-*a* showed stronger correlation with DO (positive) and Depth (negative). According to the CSTT [51] study, a seawater column with a Chl-*a* concentration value of 10 μg L^-1^ during the summer months could cause undesirable effects. In addition, Simboura et al. [16] suggested that Chl-*a* concentrations above 2.2 μg L^-1^ indicate “Bad” Ecological Status. According to the Commission Decision of 2013 [72], WFD 'high/good' thresholds for the upper 90 percentile of growing season chlorophyll in the North-East Atlantic range from 10 μg L^-1^ in the North Sea (type NEA1/26b) to 1.5 μg L^-1^ in the Spanish NEA1/26a type. The values are highly regionally specific. In the present study, Chl-*a* concentration values up to 10 μg L^-1^ have not induced even hypoxia in the oligotrophic marine environment of the Eastern Mediterranean. On the contrary, the DO concentrations had rather increased in the samples taken into account, probably due to high O_2_ release from photosynthesis. In fact, the correlation of Chl-*a* (and EI) with DO was positive (p < 0.01). It should be noted that all the samples were taken from a generally oligotrophic area receiving variable nutrient concentrations. Therefore, there was significant variation in the trophic state of the seawater column (in terms of Chl-*a* or EI) but no hypoxia was detected (89% of the sampling stations with DO > 6.5 μg L^-1^) in the seawater column including the benthic boundary layer. However, the precipitating organic material may have induced severe changes in the redox regime of the underlying sediments (33% of the stations had Eh < 0 mV) and consequently significant changes in the structure of the benthic communities. The results of the present study showed that, in the given reference framework, the variables describing the trophic status of the seawater column are more important in determining macrofaunal composition than seawater depth or sediment properties, as found in previous studies [54, 73, 74]. This may be partly because when the stations were selected, extreme conditions caused by local disturbances such as discharge of organic material [55] and trawling fisheries [56], which are known to deteriorate the Ecological Status but are not related to the sedimentation of phytoplankton biomass, were avoided.

The correlation between seawater column variables and benthic indices is useful as it can provide thresholds for Ecological Status assessment in the context of the WFD. Benthic indices are known to be significantly correlated [28, 34, 36, 37, 40] and, in addition, it has been shown by Karakassis et al. [40] that there is little change in their values when sampling methodologies (sieve mesh size, sampler size and sampling season) vary. Setting reference conditions for assessing ecological status is a major challenge in aquatic management [33]. The patchiness of the seabed characteristics, pressures and effects on many scales makes difficult any generalization of the use of indicators and their reference levels [70]). In addition, not all indicators have equally high discriminatory power or robustness to assess changes across areas with different habitats and disturbance regimes. However, it is worth mentioning that despite these shortcomings expert judgment is likely to result in very similar conclusions among experts from different geographic areas and with experience in different habitat and disturbance types [69].

The issue of Good Environmental Status has been discussed in several papers (e.g., [33, 75, 76]). The MSFD presents major challenges and opportunities for the practical use of indicators and their underlying scientific information content in supporting a balance of sustainable use of marine ecosystems for sustainability and economic prosperity [75]. The results of the present study indicate that there is a threshold in which a regime shift in the benthic community occurs as the strong coupling between “Bad” and “Poor” Ecological Status on both benthos and seawater column, implies that eutrophication may well be the driving force that changes the structure of benthic communities (Figs 4 and 5). In Fig 5, all stations with those two Ecological Status categories in benthos are located within the ellipse surrounding “Bad” or “Poor” of the seawater column. Additionally, Fig 4 indicates that samples with “Bad” and “Poor” (in most cases) are closely related with eutrophication variables. It appears that this is a threshold point after which the benthic system loses its resilience. Tett et al. [77] argue that according to the systemic approach, the persistence of an open system depends on the maintenance of its functional integrity whilst processing throughputs of energy and materials. These authors suggest that resilience is the ability to maintain integrity despite changes in boundary conditions. Additionally, Rice et al. [75] argued that communities with Good Environmental Status are those with a few abundant species and many rare ones. Such communities show a high resilience potential in the face of moderate pressures because biodiversity buffers ecosystem processes and, through these processes, the ecosystem services can be used sustainably (the complementarity hypothesis) [78]. Taking into account those arguments, strong coupling between seawater column and benthic variables in “Bad” and “Poor” environmental conditions is expected; while in “Moderate” and “Good”, other parameters such as TOC or sediment type are likely to define the structure of the benthic community.

Although threshold values are likely to be regionally valid, and seawater-body-type, specific, there is a clear need for work relating an increase of the seawater column chlorophyll concentrations to the benthic impact. The results of the present study indicate that there is a strong quantitative link between the seawater column variables and the indices describing the benthic diversity and community composition. In the context of environmental monitoring, this correlation seems to be promising in defining “high risk areas” where more intense sampling is needed, based on an initial screening of large marine areas by means of quick analysis of seawater samples. Although the results did not reveal a threshold for a “safe” ecological status for EI or Chl-*a* that could be used as an indubitable Environmental Quality Standard, it seems that the EI threshold level of 0.85, separating “Bad and Poor” from “Moderate to High” conditions [2], could serve as an acceptable critical value, above which there is a readily observable regime shift leading to a deterioration in benthic community composition.

## Supporting Information

S1 Table Data sets used in the present study. (DOCX)
