# Supplementary material for: One Step forward: Benthic Pelagic Coupling and Indicators for Environmental Status
Source: PLoS One. 2015 Oct 23;10(10):e0141071. doi: 10.1371/journal.pone.0141071 (PMC4619684; doi:10.1371/journal.pone.0141071)
Supplement: S1 Table — (DOCX) [file pone.0141071.s001.docx]

| **Serial Number** | **Location** | **Sampler** | **Sediment type** | **Depth range / Depth Average** | **Sampling year** |
| --- | --- | --- | --- | --- | --- |
| 1-21 | Malia Bay | van Veen | Mud/Sand/Silt | 10-70 / 30 | 1992–1993 |
| 22-37 | Cephalonia, Sounio, Ithaki | VanVeen | Mud/Sand/Silt | 10-40 / 30 | 1995-1997 |
| 38-56 | Evoikos gulf, Chios, Lesvos | VanVeen | Mud/Sand/Silt | 60-70 / 65 | 2001-2002 |
| 56-60 | Cephalonia, Sounio | Corers | Sand/Mud | 18-22 / 20 | 2006-2007 |
| 61-77 | Gialova bay | VanVeen | Sand/Mud | 5-10 / 6.5 | 1995-1996 |
| 78-83 | Corfu | VanVeen | Sand | 40 / 40 | 1990-1991 |
| 84-87 | Messiniakos gulf | Ponar grab | Sand/Mud | 44-52 / 51 | 2006 |
| 88-96 | Saronikos gulf | Ponar grab | Sand/Mud | 20-90 / 64 | 2004 |
| 97-102 | Saronikos gulf | Ponar grab | Sand/Mud | 20-75 / 50 | 2007 |
| 103-109 | Malliakos gulf | Ponar grab | Sand/Mud | 15-20 / 17 | 1994 |
| 110-116 | Thermaikos gulf | Ponar grab | Mud | 17-21 / 19 | 1996 |
| 117-120 | North Evoikos gulf | Ponar grab | Sand/Mud | 20-40 / 25 | 1997 |
| 121-126 | Thermaikos gulf | Ponar grab | Mud | 17-21 / 19 | 2003 |
